# Long-Aged Parmigiano Reggiano PDO: Trace Element Determination Targeted to Health

**DOI:** 10.3390/foods11020172

**Published:** 2022-01-10

**Authors:** Cristina Santarcangelo, Alessandra Baldi, Roberto Ciampaglia, Marco Dacrema, Alessandro Di Minno, Valentina Pizzamiglio, Gian Carlo Tenore, Maria Daglia

**Affiliations:** 1 Department of Pharmacy, University of Naples Federico II, 80131 Naples, Italy; cristina.santarcangelo@unina.it (C.S.); roberto.ciampaglia@unina.it (R.C.); marco.dacrema@unina.it (M.D.); alessandro.diminno@unina.it (A.D.M.); giancarlo.tenore@unina.it (G.C.T.); 2 Tefarco Innova, National Inter-University Consortium of Innovative Pharmaceutical Technologies, 43124 Parma, Italy; alessandra.baldi.alimenti@gmail.com; 3 CEINGE-Biotecnologie Avanzate, University of Naples Federico II, 80131 Naples, Italy; 4 Consorzio del Formaggio Parmigiano Reggiano, 42124 Reggio Emilia, Italy; pizzamiglio@parmigianoreggiano.it; 5 International Research Center for Food Nutrition and Safety, Jiangsu University, Zhenjiang 212013, China

**Keywords:** Parmigiano Reggiano PDO cheese, Atomic Absorption Spectroscopy, selenium, chromium, long-ripened cheese, European health claim

## Abstract

The concentrations of four health-related trace elements were measured using Atomic Absorption Spectroscopy in long-ripened (24- and 40-months) Parmigiano Reggiano (PR) PDO cheese, obtained from both summer and winter milk. To date, there are limited data on PR trace element concentrations, and no data about long-ripened cheese, especially when ripened for 40 months. Thus, the aim of this investigation is to determine chromium, manganese, selenium, and zinc concentrations, improving the available data on these trace elements and increasing knowledge of the biological properties of PR linked to their content in this cheese. The results show that 40-month ripened PR is a source of selenium and chromium, according to definitions under the European Regulation 1924/2006, as a 30 g cheese portion contains 11 ± 2 μg (summer milk) and 10 ± 1 μg (winter milk) of selenium and 8 ± 1 μg (summer and winter milk) of chromium, providing in excess of 8.25 and 6 μg per portion, respectively. This represents 15% of nutrient reference intake values for adults. These findings allow for the claim to be made that PR possesses the health properties ascribed to food sources of selenium and chromium according to European Regulation 432/2012.

## 1. Introduction

Parmigiano Reggiano cheese (PR), an Italian Protected Designation of Origin (PDO) product, is known worldwide due to its sensorial and nutritional characteristics. Furthermore, despite the product category to which it belongs, it is often associated with health, primarily due to its high protein (32.4 g/100 g), calcium (1155.0 mg/100 g), and phosphorus (691.0 mg/100 g) content, with a lower fat content (29.7 g/100 g) compared to other aged cheese, and a natural absence of lactose (less than 1.0 mg/100 g) [1].

According to the specifications currently in vigor, PR is a hard, cooked, slow-ripening cheese produced with raw partially skimmed milk, coming from cows whose diet is mainly composed of feed from its area of origin. The production area includes the territories of the provinces of Bologna to the left of the Reno River, Mantova to the right of the Po River, Modena, Parma, and Reggio Emilia. Moreover, the milk cannot be thermally treated, the use of additives is not allowed, and all milk introduced into the dairy must comply with the product specifications of PR. The ripening time must last for at least 12 months, starting from the molding of the cheese, with an average ripening of 24 months, potentially lasting up to 60 months and beyond [2].

Typically, the composition of PR varies according to the microbiological and chemical composition of the milk, the cheesemaking technology applied, including the natural whey starter, ripening time, and the environmental conditions that arise during these processes [3]. The milk composition in turn depends mainly on species, breed, season, and animal diet [4].

The specific PR cheesemaking procedures and ripening processes, along with the characteristics of the milk due to its territory of origin, strongly determine the physical properties, the chemical composition, and, in turn, the health profile of the final product. In fact, these practices lead to a selective concentration of nutritional and bioactive components, which increase the health value of this product. Due to natural dehydration occurring during the ripening process, the protein and aminoacidic content increases, mineral concentrations change (e.g., the potassium and magnesium concentrations decrease and selenium increases), and lactose decreases in the early hours following the cheese making process and is no longer detectable at 12 months of ripening [3].

There are several research articles that address PR as a source of protein, vitamins, and minerals, especially calcium, with valuable nutritional properties [5,6,7,8]. Moreover, PR, as with other fully ripened cheeses, contains other health-related nutrients, such as fats, and minor components including bioactive peptides. The fat fraction of PR contains butyric acid (123.9 mg per 100 g of fat in the outer part of the wheel at 24 months of ripening) [9], which exerts beneficial effects in obesity, inflammation, and neurological disorders, and conjugated linoleic acid (CLA—0.26 g/100 g), which has shown several beneficial activities on cardiocirculatory and immune systems [4]. As far as bioactive peptides are concerned, they are generated by proteolysis, which takes place during the ripening process and in the digestion process in humans. Among the health properties of the bioactive peptides, the inhibition of the angiotensin converting enzyme (ACE) is the most studied, along with its subsequent anti-hypertensive activity. In this regard, some studies have been published which, starting from the in vitro simulated gastrointestinal digestion of PR, demonstrate that different bioactive peptides (i.e., ACE-inhibitors and antimicrobial), are released and are absorbed in the intestine [10,11]. A recently published in silico study found an inhibitory activity of some PR cheese bioactive peptides against enzymes mainly involved in glucose metabolism, suggesting a potential effect on glycemic parameters [12].

As described above, there are investigations present in the literature that identify compounds with high healthy values in PR, but limited data are published on the concentrations of trace elements [4,13]. These elements are present in living tissues in small amounts and are known to solve essential functions for biological performance, primarily acting as cofactor catalysts in enzyme systems, as well as acting as centers for stabilizing structures of enzymes and proteins or binding molecules on the receptor sites of the cell membrane. Among these trace elements, selenium, zinc, and manganese are directly involved in the antioxidant enzymatic systems as cofactors for a number of enzymes [14]. In particular, selenium is a cofactor of 25 selenoproteins (including glutathione peroxidases, thioredoxin reductases, thioredoxin-glutathione reductase, iodothyronine deiodinases, and selenophosphate synthetase) [15,16], and zinc and manganese are cofactors of superoxide [17,18]. Chromium, as a trivalent ion, is an essential trace element, although no symptoms of chromium deficiency have been reported [19,20]. The low-molecular-weight of chromium-binding substance (LMWCr) has been proposed to be the biologically active form of chromium, being able to activate the kinase activity of insulin receptors in a dose dependent manner and increase insulin sensitivity [21].

According to the current European regulations, at present it is only possible to claim health properties of PR based on proteins, calcium, and phosphorus content, due to the limited available data on its chemical composition. Regulation (EC) 1924/2006 provides harmonized legal standards across Member States concerning nutrition and health claims, to guarantee the effective functioning of the market and a high level of consumer protection. It applies to all foods, including cheese. The term “nutrition claim” means “any indication that states, suggests, or implies that a food possesses beneficial nutritional properties due to the caloric value it provides or does not provide, or to the nutrients or other substances it contains or does not contain”. In addition to nutrition claims, this regulation also allows for health claims, as defined by Article 13 (general function claim) and Article 14 (reduction of disease risk claim). Specifically, a “health claim” is “any indication that affirms, suggests, or implies the existence of a relationship between a category of food, a food or one of its components and health, while, a “reduction of disease risk claim” defines claims relating to the reduction of a disease risk as any health claim that states, suggests or implies that the consumption of a food category, food or one of its constituents significantly reduces a risk factor of development of a human disease”.

The Regulation (EU) 432/2012 contains a list of health claims permitted for food products, which includes all 222 functional claims currently approved for description of the health properties of a food.

Considering the widespread consumption of PR, especially in Europe, the aim of this investigation is to determine the chromium, selenium, zinc, and manganese concentrations of 24-month and 40-month ripened PR, to improve the knowledge of the concentrations of these compounds and thus of the biological properties associated with the trace element content, allowing the communication of PR healthy properties to European consumers, according to Regulations 1924/2006 and 432/2012.

## 2. Materials and Methods

### 2.1. Sampling and Treatment

Cheese samples were provided by Consorzio del Formaggio Parmigiano Reggiano. A total of 100 samples of cheese (1 kg each) were randomly taken from dairies, with a proportional and representative number of samples taken from each of the 5 provinces, reflecting their proportion of the total Parmigiano Reggiano cheese wheel production. Samples were distributed according to milk production season: 25 samples of cheese aged for 24 months obtained from summer milk, 25 samples of cheese aged for 24 months obtained from winter milk, 25 samples of cheese aged for 40 months obtained from summer milk and 25 samples of cheese aged for 40 months obtained from winter milk (see detailed samples description in Supplementary Materials n. 1). Samples were chopped with a mixer (DJ3001 Moulinette Compact, Moulinex, Milano, Italy), collected in plastic tubes, and stored at −20 °C until analysis.

### 2.2. Sample Preparation

Before digestion, the cheese samples were incinerated at 500 °C for 24 h. For each cheese sample, the ash was weighed (0.5–1.5 g) and rinsed with nitric acid and three times with deionised water to remove acidic debris. For digestion, samples were transferred to TFM^®^PTFE vessels with 6 mL of concentrated 65% HNO_3_ (14.33 mol/L) and 1 mL of 30% H_2_O_2_. The samples were submitted to digestion in a microwave digestion apparatus (MW-AD, Ethos EZ microwave digester, Mileston, Shelton, CT, USA). The heating program for digestion consists of 4 steps: step 1 (90 °C for 7 min), step 2 (170 °C for 5 min), step 3 (210 °C for 5 min), and step 4 (210 °C for 20 min). In all steps the power was set to 1000 W. The final solutions were diluted up to 25 mL with doubly distilled water for analysis by Graphite Furnace Atomic Absorption Spectrometer (GFAAS).

### 2.3. Graphite Furnace Atomic Absorption Spectrometer Analysis

Analyses for Cr, Mn, Se, and Zn were performed by Atomic Absorption Spectroscopy (AAS) according to the AOAC International method, 1995. An AA-6300 atomic absorption spectrophotometer (Shimadzu, Columbia, MD, USA) was used, equipped with an ASC-6100 autosampler (Shimadzu, Columbia, MD, USA) and GFA-EX7i graphite furnace atomizer (Shimadzu, Columbia, MD, USA). The control of instruments and analysis of data were performed using Multi-Element Program Software (WizAArd software, Shimadzu, Columbia, MD, USA). Argon was used as the internal and external gas. The AAS instrument was equipped with a hollow cathode lamp for Cr, Se, Zn and Mn line sources. A deuterium lamp was used as a background corrector. Graphite pyrolytically coated tubes with a L’vov platform were employed. To optimize the analytical signal, various tests were performed with different lamp intensities, sample injection volumes and temperature ranges (1600–1800 °C for atomization).

### 2.4. Reagent and Calibration Curves

The water used was 18 megohm water, purified with a Milli-Q^®^ Integral 10 system (Merck, Darmstadt, Germany).

A selenium standard solution of 1000 mg/L, and a multi-element standard solution IV (at the concentration of 1000 mg/L): Ag, Al, Ba, Bi, Ca, Cd, Co, Cr, Cu, Fe, Ga, In, K, Mg, Mn, Na, Ni, Pb, Sr, Tl, Zn, were purchased from Merck KGaA (Darmstadt, Germany).

Calibration blank (Cal Blk) and calibration standard (Cal Std) solutions were prepared at concentrations of 0 μg/L (Cal Blk), 4 μg/L (Cal Std 1), 12 μg/L (Cal Std 2), and 20 μg/L (Cal Std 3), using the selenium standard stock solution, or multi-element standard stock solution, into separate 50 mL DigiTUBE^®^ tubes (SCP SCIENCE, Baie-D’Urfe, QC, Canada) with the addition of 0.5 mL internal standard stock solution, 0.5 mL methanol and 5 mL concentrated nitric acid, and then diluted to the final volume with water.

### 2.5. Statistical Analysis

Statistical analysis was performed to determine the differences in the obtained values between different aged PR cheeses (i.e., 24-month and 40-month ripened PR) and in different seasons (i.e., summer or winter milk). It was conducted using GraphPad Prism version 6.0.0 for Windows (GraphPad Software, San Diego, CA, USA). The results have been given in the form of mean ± SD, with *p* < 0.05 taken as statistically significant. Statistical significance of data was assessed through a one-way variance analysis (ANOVA) using Prism Graphpad 8 (San Diego, CA, USA). When significant differences were found, Tukey’s multiple comparisons test was used to determine the difference between the groups involved.

## 3. Results

Five calibration curves were prepared using standard references of the selected elements. Table 1 reports the wavelength, slit width, limit of detection (LOD), limit of quantification (LOQ), the equation of the calibration curve, the linear range, and the regression coefficient for each element. The regression coefficients obtained were greater than 0.99, except for that calculated for determination of Zn, which was found to be higher than 0.94 (Table 1). The limits of detection (LOD) and the limits of quantification (LOQ) were respectively calculated to be three times and 10 times the signal of the blank.

According to Italian LARN (Livelli di Assunzione di Riferimento di Nutrienti ed energia—Reference Intake Levels of Nutrients and Energy) a standard portion of ripened cheese is 50 g. Considering the high nutritional value of 40-month ripened PR, a portion could be quantified as 30 g. Therefore, Table 2 reports the results expressed as average value and standard deviation (SD) of the selected trace element concentrations in 100 g and in a portion of 50 g for 24-month ripened PR, and in a portion of 30 g for 40-month ripened PR.

Average chromium concentration in cheese samples obtained with summer milk and aged 24 months was found to be 13.93 ± 4.80 µg/100 g, while in cheese samples obtained with winter milk aged 24 months, the concentration was 14.61 ± 3.44 µg/100 g. For samples aged 40 months, Cr was present at higher concentrations equal to 25.34 ± 2.59 µg/100 g for summer milk samples and 26.19 ± 4.12 µg/100 g for winter milk samples. Tukey’s multiple comparisons test (Table 2) showed a statistically significant difference between samples aged 24 months and samples aged 40 months.

Average manganese concentration in cheese samples obtained with summer milk and aged 24 months resulted to be 15.96 ± 4.60 µg/100 g, while in cheese samples obtained with winter milk aged 24 months, the concentration was 7.64 ± 7.11 µg/100 g. For samples aged 40 months, Mn was present at higher concentrations equal to 40.24 ± 16.09 µg/100 g for summer milk samples and 24.96 ± 11.95 µg/100g for winter milk samples. Comparison tests showed a statistically significant difference between all groups (Table 2).

Average selenium concentration found in cheese aged 24 months from summer milk was 25.27 ± 5.82 µg/100 g, and from winter milk was 25.82 ± 2.97 µg/100 g. In cheese aged 40 months the Se concentration was equal to 37.34 ± 6.86 µg/100 g from summer milk samples, and 34.22 ± 4.26 µg/100 g from winter milk samples. Here too, the statistical analysis determined statistically significant differences between the samples aged at 24 months and samples aged at 40 months.

Average zinc concentration found in cheese aged 24 months made from summer milk was 3196 ± 1095 µg/100 g, and from winter milk it was 1752 ± 627 mg/100 g. Contrastingly, in cheese aged 40 months the Zn concentration was equal to 3952 ± 1330 µg/100 g in the summer milk samples, while it came to 2478 ± 772 µg/100 g from winter milk samples. Statistically significant differences were found in Zn concentration between groups: cheese aged 24 months, summer milk vs. cheese aged 40 months, summer milk; cheese aged 24 months, summer milk vs. cheese aged 24 months, winter milk; cheese aged 40 months, summer milk vs. cheese aged 24 months, winter milk; cheese aged 40 months, summer milk vs. cheese aged 40 months, winter milk. No statistical differences were found between the groups: cheese aged 24 months, summer milk vs. 40 months, winter milk; cheese aged 24 months, winter milk vs. cheese aged 40 months, winter milk. (Table 2).

## 4. Discussion

In this study, the concentrations of four healthy trace elements were determined in a large number of PR PDO samples with two different degrees of ripening, 24 and 40 months, taking into account different milk production seasons (summer and winter) and the percentage distribution of wheels produced among provinces of the geographical area of origin.

Trace and major elements are included in milk and cheese in a colloidal or aqueous phase depending on their type. The colloidal phase consists of proteins, caseins, organized as micelles, which during the coagulation process include the fat globules forming the curd. The aqueous phase consists of whey, which includes the soluble protein fraction, as well as monomers, small polymers, and the majority of the sugars. In the colloidal phase of milk, the casein micelles are made up of casein submicelles, cross-linked together by calcium and phosphorus, in the form of colloidal calcium phosphate (CCP). Depending on the dominant phase, colloidal or soluble, in which major minerals and trace elements can be found in milk, these can wind up in either whey or curd during the cheesemaking process. During the cheesemaking process, in the case of coagulation via lactic acid, the micelles are destabilized by acidic conditions (pH values near the isoelectric point of casein) which cause the loss of saline components including Ca and P, leading to dissociation in submicelles, which are then regrouped due to hydrophobic interaction, but not in micellar form, with Ca and P solubilized in the aqueous phase. In the case of rennet coagulation, the micelles are destabilized by rennet enzymes that cut k-casein, leading to the aggregation of many different micelles thanks to hydrophobic bonds. In this case the micelle structure is maintained, with Ca, P, and many other minerals and trace elements present in the colloidal phase (CCP). The minerals not associated with CCP (i.e., Na, and K), are almost completely lost in the whey phase, unlike the CCP minerals, such as S, Mg, and Zn, that are largely retained in curd as structural components [22]. Different factors (pH, temperature, and salinity) characterize the different cheesemaking processes, and thus play a key role in the colloidal-soluble form equilibrium of minerals and trace elements. Lactic acid fermentation leads to a decrease in milk pH, which throws off the equilibrium of the soluble form, causing a progressive solubilization of the casein-bounded minerals, losing them into the whey [23].

On the other hand, the higher temperatures promote casein-bound mineral forms. The different conditions of different cheesemaking processes lead to a variation in the concentrations of minerals and trace elements across the different kinds of cheese, despite having the same ripening period [22]. According to the literature data, the concentration of minerals with a predominantly colloidal form decreases in semi-cooked cheeses, compared to uncooked cheeses, and to lactic acid coagulation cheeses [22].

PR coagulation involves both lactic acid and rennet coagulation, with the latter being predominant. Thus, PR should be characterized by a higher concentration of minerals and trace elements that are mainly found in the colloidal casein-bound form, such as Ca, P, Zn, Se, and Cu, compared to other types of cheese, due to the predominant rennet coagulation and the long ripening process.

In addition to the cheesemaking process, cheese ripening is an important factor that influences the chemical composition of cheese, being a complex process that involves physical, chemical, and microbiological modifications, including the diffusion of salt from external to inner parts and consequent aqueous phase loss, gradual lactose loss mainly through lactic bacteria fermentation, and lactic acid neutralization leading to a pH increase. In the case of PR cheese, lactose is fully processed within 12 h post-production [24]. During the cheese ripening, minerals are progressively concentrated into the colloidal phase, due to the loss of the aqueous phase and to pH changes [25]. In addition, the basic pH environment promotes the retention of minerals in the colloidal phase. The water content of a 12-month ripened PR is 30%, decreasing to 28% in a 40-month cheese [26].

The results of our investigation show statistically significant differences between the trace element mean concentrations in 40 month-ripened PR and in 24 month-ripened PR, registered for all elements. The milk’s season of origin, however, does not influence the concentrations of the studied trace elements, with the exception of the concentration of Mn and Zn in 40 and 24 month-ripened PR, which were found to have lower concentrations in cheese obtained from winter milk. This result is due to several concurrent causes, especially humidity loss and pH changes.

At the end of 1990, Gambelli et al. [14] published a paper on minerals and trace minerals in Italian dairy products, giving a healthy connotation to trace elements for the first time. These were previously considered to be toxicological components, such as heavy metals. The research group used ion exchange liquid chromatography with suppressed conductivity for the determination of the major minerals (Na, K, Mg, and Ca) and instrumental neutron activation analysis for the determination of the trace elements (Co, Cr, Fe, Rb, Se, and Zn). The results identified two subgroups within cheeses, the stirred curd group and the hard group, both being foods with high levels of nutritionally important trace elements (i.e., Se, Zn, Fe, and Co). In particular, the hard cheese analyzed, Grana Padano (1–2 years ripened cheese samples), yielded the following concentrations: Zn 4.50 + 0.00 mg/100 g, Se 10.00 + 1.03 μg/100 g, Cr 9.90 + 2.00 μg/100 g [13], in which the concentration of Zn is similar to that found in PR, while the concentrations of Se and Cr are lower in Grana Padano than in PR according to the results of the present study.

More recently, Manuelian et al. [4] published a paper regarding major and trace elements, fatty acid composition and cholesterol content of different types of PDO cheese, including PR. Mineral concentration was measured by inductively coupled plasma optical emission spectrometry. In PR ripened for a period ranging from 12 to 24 months, Zn concentration was 33.93 ± 2.30 mg/g, and Se concentration was 0.91 ± 0.13 μg/g [4]. This concentration of Zn is similar to that found in the present investigation, and so, our results are in line with those of Manuelian and colleagues. On the contrary, the concentration of Se found by Manuelian et al. is about four times higher than the one we found, and is reported by CREA Italian Council (Consiglio per la Ricerca in agricoltura e L’analisi dell’ Economia Agraria—Council for Research in Agriculture and the Analysis of Agricultural Economics) [27].

**Figure 1:**
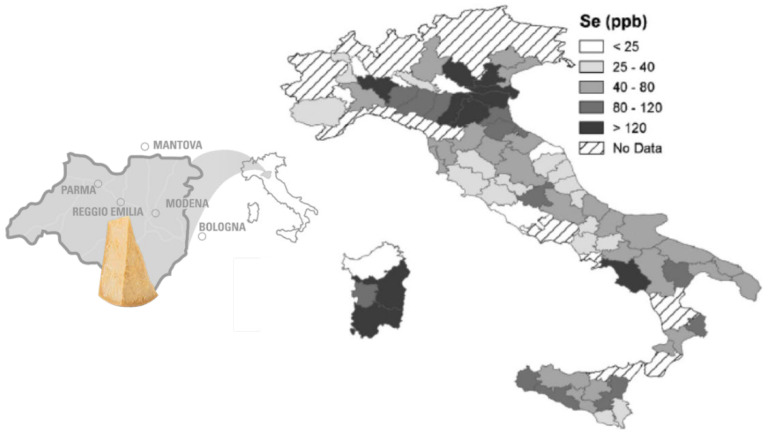
PR production area and selenium content data in Italian soil (modified from Spadoni et al., 2007) [28].

The selenium concentration reported by Manuelian actually seems to be very high, as a portion of PR alone (50 g) would provide about 45 μg, satisfying more than 82% of the Dietary Reference Values for selenium. In any case, the concentration of Se found in PR in this investigation, compared to other long-ripened cheeses, could be ascribed to the specific product PDO regulation. It requires a rationing of dairy cows based on the use of fodder from the production area of PR, and at least 50% of the dry matter of said fodder must consist of hays. Interestingly, an agronomical and geological study published in 2007 has shown that the cheese production area corresponds to a one of the three geographically separate soils richer in selenium within the Italian peninsula (Figure 1) [28]. It is well established that the selenium content of soil affects the amounts of selenium in the plants that animals eat. Nevertheless, the selenium concentration in soil has a smaller effect on trace element levels in animal products than in plant-based foods, owing to the homeostatic mechanisms present in animals and their effect on the maintenance of selenium tissue concentrations.

At the level of the European Union, as previously reported, the Regulation (EC) 1924/2006 harmonizes the laws of the Member States concerning nutrition and health claims. Generally speaking, a detailed chemical characterization of a food might be a useful approach for producers and consumers to communicate and understand the evidence-based health properties of a food.

Regarding the daily nutrient reference intakes values (NRVs) for adults, reported in annex 13 of the Regulation (EU) 1169/2011, a 30 g portion of 24-month PR could satisfy about 14% of the NRV for Se, and 11% for Cr. A 30 g portion of 40-month PR could satisfy more than 19% for both Se and Cr, making PR ripened for 40 months a confirmable source of Se and Cr, as it contains much more than the required 15% of the nutrient reference values per portion and thus easily per 100 g of food product. As far as Mn and Zn are concerned, long-aged PR contributes only a very small of the NRV for Mn. However, regarding Zn, our results showed a significant contribution towards NRV levels but only at the 100 g level, too much for a single portion consumption.

**Figure 2:**
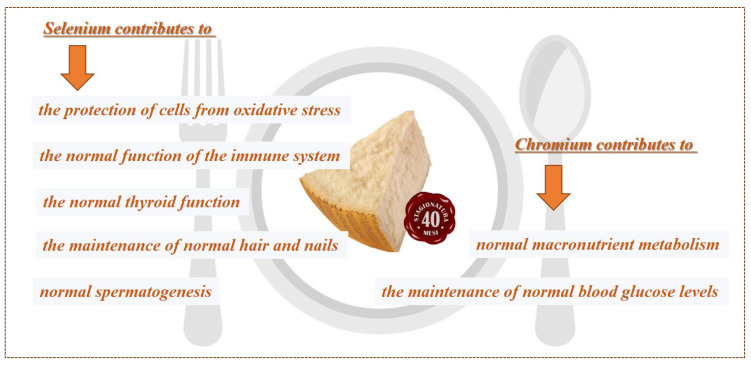
Health claim for selenium and chromium, according to the Regulation (EU) 432/2012.

These findings allow claims to be made for PR for the health properties ascribed to the food sources of selenium and chromium, according to the Regulation (EU) 432/2012 concerning health claims (Figure 2).

The most studied property of selenium is its ability to protect DNA, proteins, and lipids from oxidative damage, through its role as a cofactor for antioxidant enzymes, such as gluthation peroxidase [29]. Selenium plays a key role in both thyroid function, through the selenoproteins involved in deiodination of thyroid hormones, and in the immune system, being able to stimulate the proliferation of T cells, increase the activity of natural killer cells and the response to antigen stimulation. In addition, selenium is an important factor in spermatogenesis, as the selenoproteins of the sperm mithocondrial capsule exert structural and enzymatic functions being responsible for the motility and structural integrity of the sperm tail [30]. Finally, a deficiency in selenium, shown in patients receiving total parenteral nutrition lacking selenium, results in impairment of hair and nails, with the clinical manifestation of white nail beds, pseudoalbinism, alopecia and thin hair, which disappeared after the administration of selenium [31]. A correct intake of selenium is considered important for the maintenance of normal hair and nails.

If a correct selenium intake leads to the above reported health benefits, a selenium intake much higher than the NRV induces acute selenium toxicity, which can cause severe gastrointestinal and neurological symptoms, acute respiratory distress syndrome, myocardial infarction and, in rare cases, death, as has been observed in cases of misformulated over-the-counter products containing excessive amounts of selenium. In 2008, the US National Institute of Health (NHI) reported that 201 people experienced severe adverse reactions from taking a dietary supplement containing 200 times the labelled amount [32].

The fixed selenium upper intake level (UL) from food and supplements in the adult population is 400 µg. However, a recently published observational cohort study in a diabetes-free Italian population found that a daily quantity of Selenium equal or higher to 80 µg/day is positively associated with hospitalization for type 2 diabetes [33]. In this regard, as a selenium source, long-aged PR ripened for 40 months is a useful option for nutritionists looking to give the correct daily amount of this trace element to the general healthy population, without resorting to food supplements which remain the best option for selenium deficient subjects.

As far as chromium is concerned, this occurs in nature in the forms of trivalent and hexavalent chromium. This former form is found in various foods, including cheese, reaching higher concentrations in bivalve mollusks and Brazil nuts [19]. The latter form of chromium may be found in foods as a toxic contaminant released by the tools used in the production process (e.g., certain types of stainless steel) [34]. Trivalent chromium, taken with the diet, has positive health effects in the body [35]. Adverse reactions related to chromium deficiency have been highlighted in patients undergoing parenteral nutrition for extended periods without supplementation of this element, resulting in impaired glucose tolerance and an altered metabolism [36]. In particular, trivalent chromium is linked with an increase in insulin action and an improvement in glucose tolerance in type 2 diabetes [37]. In addition to the glucose metabolism, chromium also influences nitrogen and lipid metabolism, as it inhibits the hepatic enzyme HMG-CoA reductase and lowers LDL cholesterol levels [38]. Numerous investigations have demonstrated a relationship between the consumption of dairy products and a reduction in the occurrence of diabetes. This property of dairy products, especially PR, is generally ascribed to the protein content and the type of fats. Further studies should be conducted to identify the role of chromium in the protective effects of PR against diabetes and metabolic syndrome.

Regarding the safety of trivalent chromium, it is safe to the point that the Food and Nutrition Board of the USA National Academies of Sciences, Engineering, and Medicine has stated that no adverse effects have been linked to high intakes of chromium from food or supplements, and so it did not establish a UL for chromium [34]. In fact, no case of toxicity is recorded by the intake of trivalent chromium with food, and the only negative effects recorded are in isolated case reports of misformulated chromium supplements, which might cause weight loss, anemia, thrombocytopenia, liver dysfunction, renal failure, rhabdomyolysis, dermatitis, and hypoglycemia [39,40].

## 5. Conclusions

In conclusion, the present research work established that a portion of long-aged PR at 40-months of ripening is a source of selenium and chromium, according to the definition given by the Regulation (EC) 1924/2006, thus allowing the communication through labelling of properties typical for a food source of these trace elements to European consumers.

## Figures and Tables

**Table 1:** Analytical parameters of atomic absorption spectrometer analysis.

Element	Wavelength (nm)	Slit Width (nm)	LOD (mg/L) *	LOQ (mg/L) *	Calibration Curve	LR *	R2 *
Cr	357.9	0.7	0.0001	0.0003	y = 0.0089x + 0.0008	0–20	0.9911
Se	196	0.7	0.0001	0.0003	y = 0.0097x − 0.0008	0–20	0.9981
Zn	213.9	0.2	0.001	0.003	y = 0.0447x + 0.0949	0–20	0.9443
Mn	279.5	0.2	0.005	0.015	y = 0.0809x + 0.0104	0–20	0.9991

* LOD, limit of detection; LOQ, limit of quantification; LR, linear range; R2 regression coefficient.

**Table 2:** Concentration of trace elements in Parmigiano Reggiano cheese at different months of ripening, expressed per 100 g and per consuming portion (30 g and 50 g).

Metal	Cheese Aged 24 Months, Summer Milk *		Cheese Aged 24 Months, Winter Milk *		Cheese Aged 40 Months, Summer Milk *		Cheese Aged 40 Months, Winter Milk *	
	(µg/100 g)	(µg/50 g)	(µg/100 g)	(µg/50 g)	(µg/100 g)	(µg/30 g)	(µg/100 g)	(µg/30 g)
Cr	13.93 ± 4.80(a)	7.00 ± 2.40	14.61 ± 3.44(b)	7.30 ± 1.72	25.34 ± 2.59(a)	7.60 ± 0.77	26.19 ± 4.12(b)	7.86 ± 1.23
Mn	15.96 ± 4.60(a)	7.98 ± 2.30	7.64 ± 7.11(b)	3.82 ± 3.55	40.24 ±16.09(c)	12.07 ± 4.83	24.96 ± 11.95(d)	7.48 ± 3.58
Se	25.27 ± 5.82(a)	12.63 ± 2.91	25.82 ± 2.97(b)	12.91 ± 1.48	37.34 ± 6.86(a)	11.20 ± 2.06	34.22 ± 4.26(b)	10.27 ± 1.28
Zn	3196 ± 1095(a)	1598 ± 547.50	1752 ± 627(b)	876 ± 313.50	3952 ± 1330(c)	1186 ± 399	2478 ± 772(a,c,d)	743 ± 231

* (n = 25, ±SD); different letters indicate statistically significant differences (*p* < 0.05) between four groups (24 summer: cheese aged 24 months obtained from summer milk; 40 summer: cheese aged 40 months, summer milk; 24 winter: cheese aged 24 months, winter milk; 40 winter: cheese aged 40 months, winter milk).

## Data Availability

The data presented in this study are available on request from the corresponding author.

## Supplementary Materials

The following supporting information can be downloaded at: https://www.mdpi.com/article/10.3390/foods11020172/s1, Table S1: Selenium were determined in different cheese samples for aged and collection season milk; Table S2: Chromium were determined in different cheese samples for aged and collection season milk; Table S3: Zinc were determined in different cheese samples for aged and collection season milk; Table S4: Manganese were determined in different cheese samples for aged and collection season milk.
