# Supplementary material for: Long-Aged Parmigiano Reggiano PDO: Trace Element Determination Targeted to Health
Source: Foods. 2022 Jan 10;11(2):172. doi: 10.3390/foods11020172 (PMC8774863; doi:10.3390/foods11020172)
Supplement: Supplementary file 1 [file foods-11-00172-s001.zip › foods-1508932-supplementary.pdf]

Table S1: Selenium were determined in different cheese samples for aged and collection season milk.

Cheese aged for 24 months			
Season of milk	N° sample	µg/100g	µg/50g
Summer	1	28.80	14.40
Summer	2	29.40	14.70
Summer	3	24.00	12.00
Summer	4	33.60	16.80
Summer	5	28.80	14.40
Summer	6	20.40	10.20
Summer	7	33.60	16.80
Summer	8	21.00	10.50
Summer	9	30.00	15.00
Summer	10	25.20	12.60
Summer	11	22.80	11.40
Summer	12	21.60	10.80
Summer	13	31.20	15.60
Summer	14	21.60	10.80
Summer	15	24.00	12.00
Summer	16	18.00	9.00
Summer	17	31.20	15.60
Summer	18	15.60	7.80
Summer	19	21.60	10.80
Summer	20	26.40	13.20
Summer	21	16.80	8.40
Summer	22	14.40	7.20
Summer	23	31.20	15.60
Summer	24	27.60	13.80
Summer	25	33.60	16.80

Cheese aged for 40 months			
Season of milk	N° sample	µg/100g	µg/30g
Summer	26	33.60	10.08
Summer	27	35.40	10.62
Summer	28	33.00	9.90
Summer	29	31.80	9.54
Summer	30	35.40	10.62
Summer	31	39.00	11.70
Summer	32	40.80	12.24
Summer	33	46.80	14.04
Summer	34	43.20	12.96
Summer	35	35.40	10.62
Summer	36	43.80	13.14
Summer	37	34.80	10.44

Summer	38	28.80	8.64
Summer	39	33.60	10.08
Summer	40	46.20	13.86
Summer	41	35.40	10.62
Summer	42	37.20	11.16
Summer	43	40.80	12.24
Summer	44	34.80	10.44
Summer	45	30.60	9.18
Summer	46	56.40	16.92
Summer	47	33.60	10.08
Summer	48	28.80	8.64
Summer	49	27.60	8.28
Summer	50	46.80	14.04

	Cheese aged for 24 months		
Season of milk	N° Sample	µg/100g	µg/50g
Winter	51	28.20	14.10
Winter	52	25.20	12.60
Winter	53	30.00	15.00
Winter	54	24.00	12.00
Winter	55	27.60	13.80
Winter	56	25.20	12.60
Winter	57	26.40	13.20
Winter	58	25.20	12.60
Winter	59	30.60	15.30
Winter	60	25.20	12.60
Winter	61	25.80	12.90
Winter	62	25.20	12.60
Winter	63	27.60	13.80
Winter	64	28.80	14.40
Winter	65	25.20	12.60
Winter	66	22.80	11.40
Winter	67	19.20	9.60
Winter	68	24.00	12.00
Winter	69	21.60	10.80
Winter	70	23.40	11.70
Winter	71	28.80	14.40
Winter	72	20.40	10.20
Winter	73	28.80	14.40
Winter	74	30.00	15.00
Winter	75	26.40	13.20

	Cheese aged for 40 months		
Season of milk	N° sample	µg/100g	µg/30g
Winter	76	43.80	13.14
Winter	77	43.80	13.14
Winter	78	37.20	11.16

Winter	79	36.60	10.98
Winter	80	32.40	9.72
Winter	81	30.60	9.18
Winter	82	32.40	9.72
Winter	83	32.40	9.72
Winter	84	40.20	12.06
Winter	85	32.40	9.72
Winter	86	33.60	10.08
Winter	87	28.80	8.64
Winter	88	40.20	12.06
Winter	89	37.20	11.16
Winter	90	32.40	9.72
Winter	91	33.00	9.90
Winter	92	34.80	10.44
Winter	93	33.00	9.90
Winter	94	30.60	9.18
Winter	95	28.20	8.46
Winter	96	36.60	10.98
Winter	97	32.40	9.72
Winter	98	32.40	9.72
Winter	99	31.20	9.36
Winter	100	29.40	8.82

Table S2: Chromium were determined in different cheese samples for aged and collection season milk.

Cheese aged for 24 months			
Season of milk	N° Sample	µg/100g	µg/50g
Summer	1	12.13	6.07
Summer	2	14.11	7.06
Summer	3	9.99	5.00
Summer	4	24.11	12.06
Summer	5	15.44	7.72
Summer	6	13.77	6.89
Summer	7	21.11	10.56
Summer	8	19.77	9.89
Summer	9	11.34	5.67
Summer	10	13.28	6.64
Summer	11	7.17	3.59
Summer	12	13.15	6.58
Summer	13	14.16	7.08
Summer	14	9.21	4.61
Summer	15	7.77	3.89
Summer	16	8.91	4.46
Summer	17	15.88	7.94
Summer	18	20.11	10.06
Summer	19	17.66	8.83
Summer	20	16.99	8.50
Summer	21	16.88	8.44
Summer	22	6.22	3.11
Summer	23	6.88	3.44
Summer	24	14.11	7.06
Summer	25	18.22	9.11
Cheese aged for 40 months			
Season of milk	N° Sample	µg/100g	µg/30g
Summer	26	26.83	8.05
Summer	27	25.35	7.61
Summer	28	23.99	7.20
Summer	29	25.93	7.78
Summer	30	21.01	6.30
Summer	31	28.80	8.64
Summer	32	26.52	7.96
Summer	33	25.15	7.55
Summer	34	21.64	6.49
Summer	35	28.13	8.44
Summer	36	25.77	7.73
Summer	37	22.62	6.79
Summer	38	27.76	8.33

Summer	39	22.14	6.64
Summer	40	25.45	7.64
Summer	41	22.75	6.83
Summer	42	24.13	7.24
Summer	43	21.79	6.54
Summer	44	23.55	7.07
Summer	45	25.90	7.77
Summer	46	23.71	7.11
Summer	47	26.71	8.01
Summer	48	28.60	8.58
Summer	49	28.71	8.61
Summer	50	30.70	9.21
	Cheese aged for 24 months		
Season of milk	N° Sample	µg/100g	µg/50g
Winter	51	14.66	7.33
Winter	52	15.12	7.56
Winter	53	16.66	8.33
Winter	54	18.77	9.39
Winter	55	14.11	7.06
Winter	56	20.11	10.06
Winter	57	18.11	9.06
Winter	58	13.70	6.85
Winter	59	16.51	8.26
Winter	60	17.55	8.78
Winter	61	16.11	8.06
Winter	62	10.87	5.44
Winter	63	16.98	8.49
Winter	64	12.88	6.44
Winter	65	12.11	6.06
Winter	66	9.89	4.95
Winter	67	7.88	3.94
Winter	68	18.14	9.07
Winter	69	13.05	6.53
Winter	70	10.88	5.44
Winter	71	7.87	3.94
Winter	72	19.88	9.94
Winter	73	15.77	7.89
Winter	74	12.66	6.33
Winter	75	14.99	7.50
	Cheese aged for 40 months		
Season of milk	N° Sample	µg/100g	µg/30g
Winter	76	29.18	8.75
Winter	77	27.01	8.10
Winter	78	20.23	6.07
Winter	79	21.88	6.56

Winter	80	23.40	7.02
Winter	81	20.74	6.22
Winter	82	24.59	7.38
Winter	83	23.74	7.12
Winter	84	29.77	8.93
Winter	85	30.65	9.20
Winter	86	25.53	7.66
Winter	87	24.85	7.46
Winter	88	26.09	7.83
Winter	89	25.41	7.62
Winter	90	29.73	8.92
Winter	91	27.52	8.26
Winter	92	24.88	7.46
Winter	93	31.91	9.57
Winter	94	28.69	8.61
Winter	95	27.35	8.21
Winter	96	22.09	6.63
Winter	97	16.66	5.00
Winter	98	27.85	8.36
Winter	99	29.71	8.91
Winter	100	35.29	10.59

Table S3: Zinc were determined in different cheese samples for aged and collection season milk.

Cheese aged for 24 months			
Season of milk	N° sample	µg/100g	µg/50g
Summer	1	3.93	1.97
Summer	2	3.81	1.91
Summer	3	2.02	1.01
Summer	4	2.54	1.27
Summer	5	4.18	2.09
Summer	6	3.74	1.87
Summer	7	4.60	2.30
Summer	8	2.70	1.35
Summer	9	2.78	1.39
Summer	10	4.20	2.10
Summer	11	2.46	1.23
Summer	12	2.10	1.05
Summer	13	2.30	1.15
Summer	14	2.78	1.39
Summer	15	2.38	1.19
Summer	16	1.86	0.93

Summer	17	1.74	0.87
Summer	18	4.37	2.19
Summer	19	2.47	1.24
Summer	20	5.80	2.90
Summer	21	5.45	2.73
Summer	22	2.98	1.49
Summer	23	3.12	1.56
Summer	24	2.95	1.48
Summer	25	2.67	1.34

	Cheese aged for 40 months		
Season of milk	N° sample	µg/100g	µg/30g
Summer	26	4.49	1.35
Summer	27	5.45	1.64
Summer	28	4.83	1.45
Summer	29	3.43	1.03
Summer	30	5.71	1.71
Summer	31	5.21	1.56
Summer	32	2.84	0.85
Summer	33	2.93	0.88
Summer	34	4.50	1.35
Summer	35	4.31	1.29
Summer	36	2.45	0.74
Summer	37	4.65	1.40
Summer	38	0.13	0.04
Summer	39	4.12	1.24
Summer	40	4.11	1.23
Summer	41	3.11	0.93
Summer	42	4.11	1.23
Summer	43	3.38	1.01
Summer	44	5.90	1.77
Summer	45	2.34	0.70
Summer	46	4.76	1.43
Summer	47	2.38	0.71
Summer	48	5.66	1.70
Summer	49	3.46	1.04
Summer	50	4.55	1.37

Cheese aged for 24 months			
Season of milk	N° sample	µg/100g	µg/50g
Winter	51	1.61	0.81
Winter	52	1.88	0.94
Winter	53	1.77	0.89
Winter	54	1.56	0.78
Winter	55	1.71	0.86
Winter	56	0.99	0.50
Winter	57	1.21	0.61

Winter	58	1.23	0.62
Winter	59	1.87	0.94
Winter	60	1.66	0.83
Winter	61	1.45	0.73
Winter	62	2.09	1.05
Winter	63	1.66	0.83
Winter	64	2.13	1.07
Winter	65	2.19	1.10
Winter	66	3.60	1.80
Winter	67	1.77	0.89
Winter	68	1.41	0.71
Winter	69	0.91	0.46
Winter	70	1.77	0.89
Winter	71	1.54	0.77
Winter	72	2.66	1.33
Winter	73	3.01	1.51
Winter	74	1.02	0.51
Winter	75	1.11	0.56

Cheese aged for 40 months

Season of milk	N° sample	μg/100g	μg/30g
Winter	76	1.88	0.56
Winter	77	3.22	0.97
Winter	78	2.88	0.86
Winter	79	3.82	1.15
Winter	80	2.82	0.85
Winter	81	3.33	1.00
Winter	82	2.78	0.83
Winter	83	2.99	0.90
Winter	84	3.33	1.00
Winter	85	2.14	0.64
Winter	86	2.66	0.80
Winter	87	3.33	1.00
Winter	88	1.99	0.60
Winter	89	2.19	0.66
Winter	90	3.11	0.93
Winter	91	2.09	0.63
Winter	92	2.99	0.90
Winter	93	2.12	0.64
Winter	94	3.11	0.93
Winter	95	1.81	0.54
Winter	96	2.15	0.65
Winter	97	1.11	0.33
Winter	98	0.99	0.30
Winter	99	2.12	0.64
Winter	100	0.98	0.29

Table S4: Manganese were determined in different cheese samples for aged and collection season milk.

Cheese aged for 24 months			
Season of milk	N° sample	µg/100g	µg/50g
Summer	1	0.012	0.006
Summer	2	0.015	0.008
Summer	3	0.018	0.009
Summer	4	0.021	0.011
Summer	5	0.013	0.007
Summer	6	0.013	0.007
Summer	7	0.013	0.007
Summer	8	0.018	0.009
Summer	9	0.006	0.003
Summer	10	0.015	0.008
Summer	11	0.019	0.010
Summer	12	0.018	0.009
Summer	13	0.014	0.007
Summer	14	0.007	0.004
Summer	15	0.022	0.011
Summer	16	0.013	0.007
Summer	17	0.023	0.012
Summer	18	0.016	0.008
Summer	19	0.024	0.012
Summer	20	0.018	0.009
Summer	21	0.017	0.009
Summer	22	0.014	0.007
Summer	23	0.011	0.006
Summer	24	0.017	0.009
Summer	25	0.022	0.011
	Cheese aged for 40 months		
Season of milk	N° sample	µg/100g	µg/30g
Summer	26	0.025	0.008
Summer	27	0.022	0.007
Summer	28	0.020	0.006
Summer	29	0.019	0.006
Summer	30	0.029	0.009
Summer	31	0.068	0.020
Summer	32	0.057	0.017
Summer	33	0.036	0.011
Summer	34	0.039	0.012

Summer	35	0.040	0.012
Summer	36	0.042	0.013
Summer	37	0.076	0.023
Summer	38	0.040	0.012
Summer	39	0.032	0.010
Summer	40	0.080	0.024
Summer	41	0.047	0.014
Summer	42	0.037	0.011
Summer	43	0.046	0.014
Summer	44	0.045	0.014
Summer	45	0.038	0.011
Summer	46	0.035	0.011
Summer	47	0.036	0.011
Summer	48	0.018	0.005
Summer	49	0.040	0.012
Summer	50	0.039	0.012
Cheese aged for 24 months			
Season of milk	N° sample	µg/100g	µg/50g
Winter	51	0.006	0.003
Winter	52	0.002	0.001
Winter	53	0.004	0.002
Winter	54	0.005	0.003
Winter	55	0.004	0.002
Winter	56	0.007	0.004
Winter	57	0.008	0.004
Winter	58	0.007	0.004
Winter	59	0.003	0.002
Winter	60	0.005	0.003
Winter	61	0.004	0.002
Winter	62	0.008	0.004
Winter	63	0.007	0.004
Winter	64	0.005	0.003
Winter	65	0.006	0.003
Winter	66	0.008	0.004
Winter	67	0.003	0.002
Winter	68	0.010	0.005
Winter	69	0.006	0.003
Winter	70	0.007	0.004
Winter	71	0.040	0.020
Winter	72	0.009	0.005
Winter	73	0.007	0.004
Winter	74	0.011	0.006
Winter	75	0.009	0.005
Cheese aged for 40 months			
Season of milk	N° sample	µg/100g	µg/30g

Winter	76	0.015	0.005
Winter	77	0.011	0.003
Winter	78	0.01	0.003
Winter	79	0.011	0.003
Winter	80	0.013	0.004
Winter	81	0.028	0.008
Winter	82	0.024	0.007
Winter	83	0.026	0.008
Winter	84	0.021	0.006
Winter	85	0.012	0.004
Winter	86	0.032	0.01
Winter	87	0.055	0.017
Winter	88	0.02	0.006
Winter	89	0.028	0.008
Winter	90	0.05	0.015
Winter	91	0.037	0.011
Winter	92	0.032	0.01
Winter	93	0.038	0.011
Winter	94	0.037	0.011
Winter	95	0.018	0.005
Winter	96	0.022	0.007
Winter	97	0.025	0.008
Winter	98	0.011	0.003
Winter	99	0.025	0.008
Winter	100	0.023	0.007
